# More preoperative flexibility implies adequate neural pliability for curve correction without prophylactic untethering in scoliosis patients with asymptomatic tethered spinal cord, a retrospective study

**DOI:** 10.1186/s12891-017-1615-0

**Published:** 2017-06-15

**Authors:** Zhenhai Zhou, Hongqi Zhang, Chaofeng Guo, Honggui Yu, Longjie Wang, Qiang Guo

**Affiliations:** Department of Spine Surgery, Xiangya Hospital of Central South University, Xiangya Road 87, Changsha, Hunan Province 410008 China

**Keywords:** Scoliosis, Tethered spinal cord, Asymptomatic, Posterior scoliosis correction

## Abstract

**Background:**

Tethered spinal cord is frequently associated with scoliosis. It is still controversial whether a prophylactic untethering is necessary before correction procedure in scoliosis patients with tethered spinal cord. In this study we determined the clinical outcome of a one-stage posterior scoliosis correction without a prophylactic untethering for treating scoliosis with an asymptomatic tethered spinal cord.

**Methods:**

Seventeen (5 males and 12 females) scoliosis patients with tethered spinal cords were retrospectively reviewed. All patients underwent a one-stage posterior scoliosis correction without preventive untethering. Parameters of radiograph were used to assess correction result. The Scoliosis Research Society (SRS)-22 questionnaire was analyzed pre- and post-operatively to evaluate the clinical outcomes. The modified Japanese Orthopaedic Association (mJOA) score was used to assess the pre- and post-operative spinal cord function.

**Results:**

The post-operative coronal Cobb angle was significantly decreased compared with preoperative. (23.8 ± 6.4° vs. 58.4 ± 12.6°, *P* < 0.01). The coronal Cobb angle was 22.4 ± 6.8° at the final follow-up evaluation. The apical vertebral translation (AVT) was also decreased significantly. (27.5 mm vs. 60.9 mm, *P* < 0.01). The SRS-22 total score was improved at the 1-year follow-up evaluation compared with the pre-operative SRS-22 total score (87 ± 4 vs. 70 ± 5, *p* < 0.05). The functional activities, pain, self-image, mental health, and surgery satisfactory scores at the final follow-up evaluation were all improved compared with the corresponding pre-operative scores, especially the self-image and mental health scores (*p* < 0.05). The spinal cord function was stable and there was no new neurological symptoms after scoliosis correction. No difference existed between the pre- and post-operative total mJOA score (26 ± 2 vs. 27 ± 2, *p* = 0.39), which including subjective symptom (*p* = 0.07), clinical symptom (*p* = 0.33), daily activities (*p* = 0.44) and bladder function (*p* = 0.67).

**Conclusion:**

One-stage posterior scoliosis correction is a safe and effective surgical procedure for scoliosis patients combined with asymptomatic tethered spinal cord who have adequate spinal cord function reserve.

## Background

Scoliosis is often associated with a number of spinal cord malformations, such as tethered spinal cords, diastomyelia, lipomyelomeninggoceles, and Chiari deformities. In addition, it has been shown that 15–30% of patients with congenital scoliosis have spinal malformations [1–3]. TCS is a intraspinal anomaly, caused by abnormal spinal cord fixation and resultant low-lying and immobile conus medullaris. The incidence of TCS is estimated to be 0.05 to 0.25 per 1000 births. It may result from many causes, including intraspinal lipoma, lipomyelomeningocele, diastematomyelia, spina bifida occulta, tight or thickened filum terminale, or scarring from previous myelomeningocele repair [4]. Although rare, a tethered spinal cord is one of the most common pediatric disorders [5]. Tethered cord refers to intradural thickening of the filum, shortening or adherence to the spinal cord, and a limitation of movement between the spinal cord and spinal canal. A tethered spinal cord can be diagnosed if the conus medularis is located below the L1–2 level and/or a thickened filum terminale is present (>2 mm) on radiologic images [6]. In the process of growth and development of the spinal cord, spinal cord tension is increased, resulting in spinal cord ischemia, hypoxia, and degeneration [7]. Although tethered spinal cord is a congenital disorder which is present from birth, the symptoms of tethered cord can appear later, during the teenage years and adulthood, even no symptoms lifelong. Once symptoms developed, the patient has a tethered cord syndrome (TCS) [8]. The syndrome is usually diagnosed in childhood and the presentation is variable and may be insidious. The main clinical manifestations of TCS were numbness, weakness, and urination and defecation dysfunction. However, some patients have no neurologic symptoms [9] or no clinical symptoms and a tethered cord are found during a routine physical examination.

For scoliosis patients with a tethered cord, limited movement between the tethered cord and spinal canal greatly increases the level of difficulty in performing a scoliosis correction procedure, and also aggravates neurologic dysfunction or causes new neurologic deficits in the operative process. Traditional theory has shown that surgical detethering should be performed first, followed by scoliosis correction several months later [10, 11]; however, the scoliosis may deteriorate before the correction procedure is performed. Some studies have shown that surgical detethering and scoliosis correction can be performed concurrently and the risk of complications is not increased [12, 13]. It is still controversial whether or not preventive untethering is necessary before scoliosis correction; further studies are warranted.

In the current study, 17 scoliosis patients with asymptomatic tethered cords who underwent a one-stage posterior scoliosis correction procedure without preventive untethering were retrospectively reviewed. The coronal Cobb angle, apical translation (AVR), the curve flexibility and the correction rate (CR) were recorded and analyzed to evaluate correction result. We also analyzed the Scoliosis Research Society (SRS)-22 questionnaire, and the mJOA score pre- and post-operatively to evaluate the clinical outcomes.

## Methods

### Clinical characteristics

**Fig.1:**
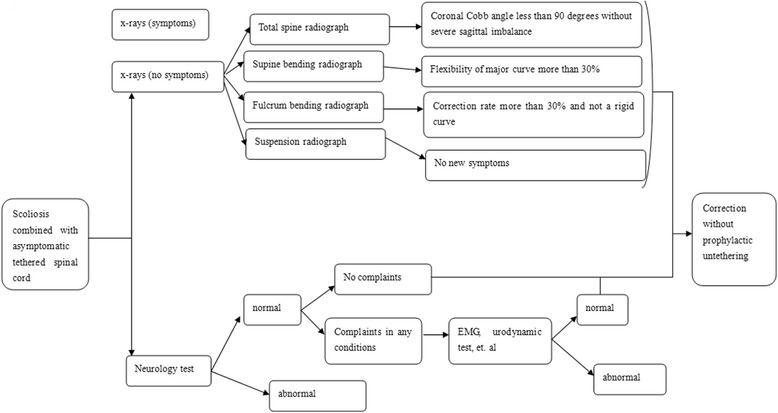
A simple flowchart of adaptability of one-stage posterior correction for scoliosis combined with asymptomatic tethered spinal cord without a prophylactic untethering. All the patients who planed to under go a surgical correction should accept rigorous neurological physical examination (including sensation, muscle strength, physiological reflexes and pathological reflexes) and imaging examination (including radiograph, CT and MRI). A tethered cord can be found with a MRI examination. The patients who had no symptoms while they underwent any position of radiograph could accept correction without a prophylactic untethering. Additionally, the patients who had complaints but with a normal related-examination, such as EMG and urodynamic test, also could accept one-stage correction without a cord release

Seventeen scoliosis patients (5 males and 12 females; mean age, 14.8 years; age range, 9–17 years) with asymptomatic tethered spinal cords were reviewed in this study. They were 14 congenital scoliosis, 2 idiopathic scoliosis and 1 neuromuscular scoliosis. The location of major curve are as following: 18% (3/17) in the proximal thoracic (PT), 47% (8/17) in the main thoracic (MT), 18% (3/17) in the thoracolumbar (TL) and 18% (3/17) in the lumbar (L). All the patients presented coronal imbalance and had no or mild sagittal imbalance. The patients who underwent any kinds of detethering before and the patients with severe kyphosis and kyphoscoliosis, who need a osteotomy for correction, were excluded from this study. All of the patients underwent one-stage posterior scoliosis correction without preventive untethering. Termination of the spinal cord in all patients was located below L3 and all of the patients underwent rigorous neurological physical examination, which including sensation, muscle strength, physiological reflexes and pathological reflexes. There was neither abnormality in physical examination nor apparent neurologic dysfunction before the scoliosis correction. Seven patients, including five patients complained of minor weakness involving the lower limbs after strenuous exercise and 2 patients complained of irregular urination, underwent additional neural electromyography and urodynamic test. Based on all normal test results, these seven patients were added to this study (Table 1, Fig. 1).

**Table 1:** Clinical characteristic of subjects

Variables	Data
Gender	
Male	5
Female	12
Age	14.8 (9–17)
Cobb angle	58.4 ± 12.6
Etiologic classification	
Congenital scoliosis	14
Idiopathic scoliosis	2
Neuromuscular scoliosis	1
Symptoms	
Pain	0 (slight paroxysmal low back pain)
Mimor weakness	5 (after strenuous excise)
Sensory dysfunction	0
Bladder dysfunction	2 (irregular urination)
Scoliosis progression	9
Leg lenth discrepancy	0
Perianal skin abnormaltities	0
Reasons of tethered cord	
lipoma	2
Spinal meningocele	1
Low-lying conus medullaris	8
Thickened filum terminale	4
Diastematomyelia	2 (type II)
Additional deformity	
Spina bifida occulta	3
Vertebral deformiy	3
Syringomyelia	2
Ribs deformity	1

### Imaging procedure

Radiographs of the total spine, three-dimensional computed tomography (CT) reconstruction, and magnetic resonance imaging (MRI) were performed pre- post-operatively. Preoperative posteroanterior radiograph, supine bending radiograph and fulcrum bending radiograph were used to assess curve flexibility and expected correction rate. Parameters of radiographs, including the coronal Cobb angles, apical vertebral translation (AVT), apical vertebral rotation (AVR), flexibility and correction rate (CR), were measured and recorded. X-ray were also performed every follow up to evaluate the correction result and observe the location of the pedicle screws. Moreover, a additional MRI was selectively used for those patients who were suspected to have developed symptoms of TCS in follow up.

Flexibility (%) = (preoperative Cobb angle-supine bending Cobb angle)/preoperative Cobb angle × 100%

Expected correction rate (%) = (preoperative Cobb angle-fulcrum bending Cobb angle)/preoperative Cobb angle × 100%

Operation correction rate (%) = (preoperative Cobb angle-postoperative Cobb angle)/preoperative Cobb angle × 100%

Final correction rate (%) = (preoperative Cobb angle-final Cobb angle)/preoperative Cobb angle × 100%

### Operative procedure

Spine suspension, supine bending and fulcrum bending roentgenograph examinations were performed in all patients; no apparent symptoms of a tethered spinal cord appeared. Based on normal physical examination and auxiliary inspections, neurosurgeons considered that an untethering was not indicated in these asymptomatic patients before a scoliosis correction procedure, but a closely follow-up was also suggested after scoliosis correction. Therefore, all of the patients underwent one-stage posterior deformity correction without a prophylactic untethering. Polyaxial screws were used for posterior instrumentation and scoliosis correction. Intra-operative somatosensory evoked potentials (SEPs), motor evoked potentials (MEPs), and wake-up tests were applied during the operation for detecting neurological function.

### Clinical evaluation

Scoliosis Research Society (SRS)-22 questionnaires and modified Japanese Orthopaedic Association (mJOA) scores were evaluated pre-operatively and at the 1-year follow-up evaluation to assess clinical result and neurologic function. The SRS-22 questionnaire functional activities, pain, self-image, and mental health scores were evaluated. The mJOA score mainly included subjective symptoms, clinical symptoms, daily activities, and bladder function.

### Follow-up

All patients were followed every 3–6 months and all patients followed for more than 1 years. The SRS-22 questionnaire, mJOA score, and radiologic imaging at the 1-year follow-up were evaluated.

### Statistical analysis

Data were managed and analyzed using SPSS 17.0. Data are shown as the mean ± SD. A paired sample t-test was used to compare the pre- and post-operative data. A *P* < 0.05 was considered statistically significant.

## Results

### Clinical result

All the patients accepted rigorous neurological physical examination after operation and in follow up time. The original seven patients who had complaints also underwent additional neural electromyography and urodynamic test. There was no new symptoms appeared after the scoliosis correction and none of the patients experienced deterioration in their neurological status after surgery. None of the patients underwent any kinds of tethered cord release until last follow up.

### Correction result

**Fig. 2:**
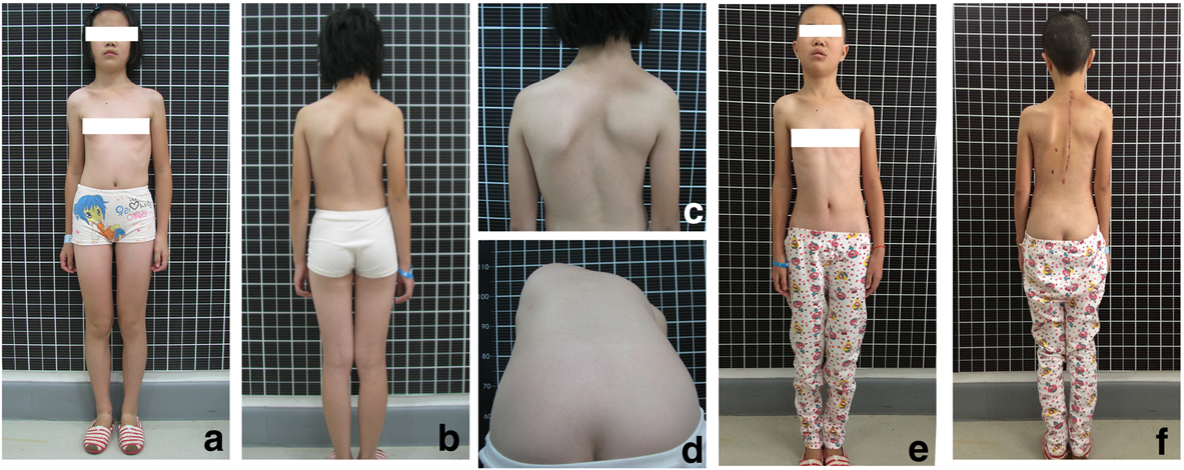
Case 5. An 11-year-old female scoliosis patient with an asymptomatic tethered spinal cord. The patient had no lower back pain, weakness of the lower limbs, or bladder dysfunction. The pre-operative photos (Fig. 3 **a**-**d**) show scoliosis with imbalance of shoulders and razor back.The post-operative images (Fig. 3 **e**-**f**) show scoliosis was well corrected

**Fig. 3:**
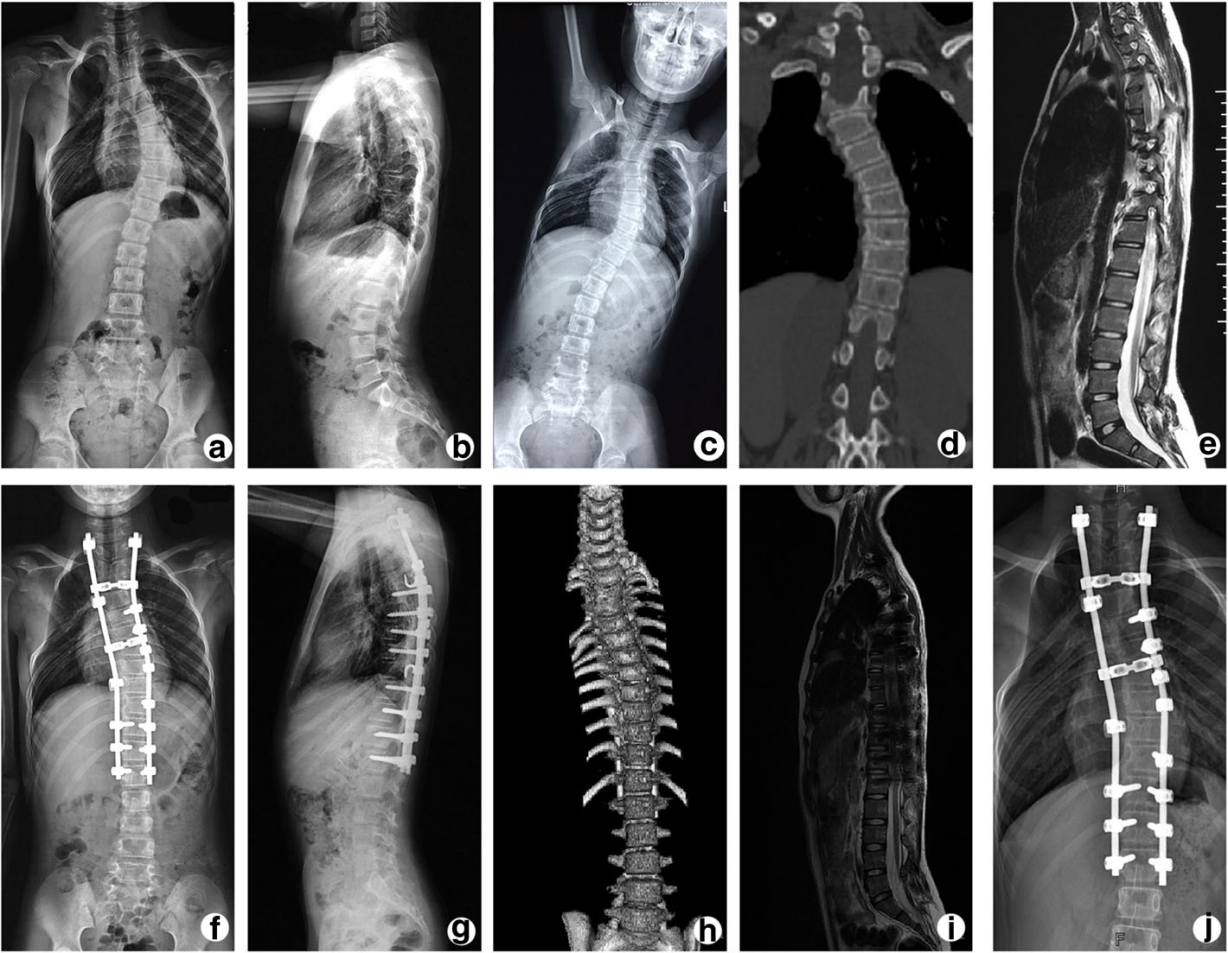
The pre-operative radiograph and CT (Fig. 3 **a**-**d**) shows scoliosis with a coronal Cobb angle of 54.0^o^. The AVT was 61mm and the flexibility was 53.7%. The pre-operative MRI (Fig. 3 **e**) shows a tethered spinal cord and the conus medularis located at the L4-L5 level. The post-operative radiographs and CT (Fig. 3 **f**-**h**) shows that the coronal Cobb angle is decreased to approximately 22^o^. The VAT decreased to 36mm and the OP correction rate was 66.7%. The post-operative MRI (Fig. 3 **i**) shows the conus medularis has no upward movement and is still located at the L4-L5 level. The radiograph (Fig. 3 **j**) at the 1-year follow-up evaluations shows no correction loss after the operation and the final correction was 66.7%

**Fig. 4:**
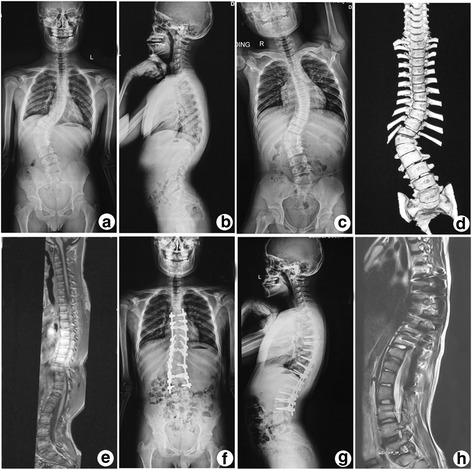
Case 7. A 15-year-old boy presented scoliosis combined with asymptomatic tethered cord. The pre-operative radiograph and CT (Fig. 2 **a**-**d**) shows scoliosis with a coronal Cobb angle of 66.0^o^. The AVT was 66 mm and the flexibility was 36.5%. The pre-operative MRI (Fig. 2 **e**) shows a tethered spinal cord and the conus medularis located at the L3 level. The final radiographs (Fig. 2 **f**, **g**) shows that the coronal Cobb angle is decreased to 23^o^. The VAT decreased to 10 mm and the correction rate was 62.9%. The post-operative MRI (Fig. 2 **h**) shows the conus medularis has no upward movement and is still located at the L3 level

In these 17 patients, the mean flexibility was 42.9% and mean expected correction rate was 51.9%. The pre- and post-operative coronal Cobb angles were 58.4 ± 12.6^o^ and 23.8 ± 6.4^o^, respectively. The operation correction rate was 58.6%. The final Cobb angle at 1 year follow up was 22.4 ± 6.8^o^ and the final correction rate was 60.9%. A significant difference existed between the pre- and post-operative Cobb angles (*p* < 0.01). The pre- and post-operative apical vertebral translation (AVT) were 60.9 ± 20.4 mm and 26.4 ± 9.0 mm, respectively. The AVT was 27.5 ± 10.2 mm at the 1-year follow-up evaluation. A significant difference existed between the pre- and post-operative AVT (*p* < 0.01). Satisfactory correction was achieved and there was no correction loss noted at the 1-year follow-up (Table 2, Figs. 2, 3 and 4).

**Table 2:** The radiographic parameters of preoperative, postoperative and follow up

Case	Sex	Age (years)	Location of the major curve	AV	AVT (mm)			AVR	Risser score	Pre-OP Cobb (°)	Bending Cobb (°)	Flexibility	Fulcrum Bending Cobb (°)	EX-CR	Post-op Cobb (°)	OP-CR	Visit Cobb (°)	Final CR
					Pre-OP	Post-OP	Final											
1	F	16	MT	T7	68	32	34	0	5	46	29	37.0%	27	41.3%	32	30.4%	30	34.8%
2	F	13	PT	T4	85	40	36	0	4	67	38	43.3%	30	55.2%	28	58.2%	30	55.2%
3	M	16	MT	T8	54	18	17	2	4	68	41	39.7%	36	47.1%	31	54.4%	36	47.1%
4	F	14	MT	T10	46	19	16	0	4	49	26	46.9%	22	55.1%	19	61.2%	21	57.1%
5	F	11	MT	T8	61	36	40	1	1	54	25	53.7%	20	63.0%	18	66.7%	18	66.7%
6	F	16	TL	T12	73	33	39	3	5	44	25	43.2%	18	59.1%	14	68.2%	14	68.2%
7	M	15	TL	T12	66	12	10	0	1	62	40	36.5%	30	51.6%	23	62.9%	20	67.7%
8	M	16	MT	T8	50	26	28	2	5	74	30	59.5%	30	59.5%	32	56.8%	32	56.8%
9	F	17	MT	T6	65	21	24	1	5	82	40	51.2%	34	58.5%	29	64.6%	25	69.5%
10	F	16	L	L2	58	20	25	0	5	54	31	42.6%	29	46.3%	20	63.0%	15	72.2%
11	F	15	TL	T12	96	34	32	2	4	78	49	37.2%	36	53.8%	30	61.5%	18	76.9%
12	M	16	L	L3	42	27	30	3	4	48	28	41.7%	28	41.7%	26	45.8%	20	58.3%
13	F	14	MT	T6	52	22	22	1	4	52	35	32.7%	28	46.2%	20	61.5%	20	61.5%
14	F	16	PT	T12	46	20	21	0	5	46	24	47.8%	24	47.8%	26	43.5%	25	45.7%
15	M	9	PT	T3	55	23	23	0	1	54	35	35.2%	31	42.6%	20	63.0%	22	59.3%
16	F	16	L	L2	45	29	33	2	5	49	32	34.7%	22	55.1%	16	67.3%	16	67.3%
17	F	15	MT	T9	74	37	38	0	5	66	36	45.5%	27	59.1%	21	68.2%	19	71.2%
AVG		14.8			60.9	26.4	27.5			58.4	33.1	42.9%	27.8	51.9%	23.8	58.6%	22.4	60.9%

(Note: *MT* Main thoracic, *PT* proximal thoracic, *TL* thoracolumbar, *L* lumbar, *AV* apical vertebrae, *AVT* apical vertebral translation, *AVR* apical vertebral rotation, *Pre-OP Cobb* preoperative Cobb’s angle, *Bending Cobb* Cobb’s angle in reverse bending film, *Flexibility* (pre-OP Cobb –bending Cobb)/pre-op Cobb, *EX-CR* expected correction rate = (pre-OP Cobb –fulcrum bending Cobb)/pre-OP Cobb, *OP-CR* operative correction rate, *Final CR* final correction rate, *AVG* = average.)

### SRS-22 score

**Fig. 5:**
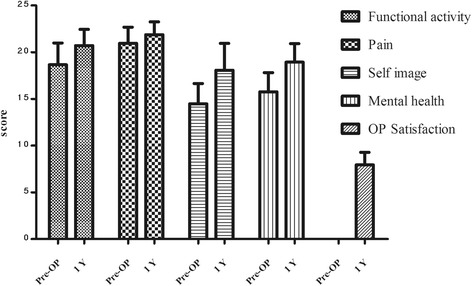
SRS-22 score pre-operative (Pre-OP) and at the 1-year follow-up evaluation (1Y) in scoliosis patients with tethered spinal cords. * *P* < 0.05 vs. pre-operative

All of the patients completed a SRS-22 questionnaire and the scores were measured pre-operatively and at the 1-year follow-up evaluation. The functional activities, pain, self-image, and mental health scores were all improved post-operatively (Table 3 and Fig. 5), especially the self-image and mental health scores (*p* < 0.05). The pre-operative total score was 70 ± 5, and the score increased to 87 ± 4. A significant difference existed between the pre-operative and follow-up evaluation scores (*p* < 0.01).

**Table 3:** SRS-22 score of preoperative and 1 year follow up

Parameters	Preoperative	1 year follow up	T value	*P* value
Functional activity	17 ± 2	21 ± 2	−3.38	0.00
Pain	21 ± 2	22 ± 1	−2.99	0.01
Self image	14 ± 2	18 ± 2	−5.15	0.00
Mental health	16 ± 2	19 ± 2	−4.62	0.00
OP Satisfaction	----------	8 ± 1	----------	----------
SRS-22 total score	70 ± 5	87 ± 4	−11.47	0.00

(Note:SRS-22 questionnaires including five aspects: 1 Recovery of functional activities of patients include quesetion 5、9、12、、15、18; 2. Improvement of pain of the patients include question 1、2、8、11、17;3. Assessment of self image of the patients include question 4、6、2、14、19;4 Assessment of mental health of the patients include question 3、7、13、16、20; 5 Operation satisfaction was only answered by patients performed operation include question21、22.)

### mJOA score

**Fig. 6:**
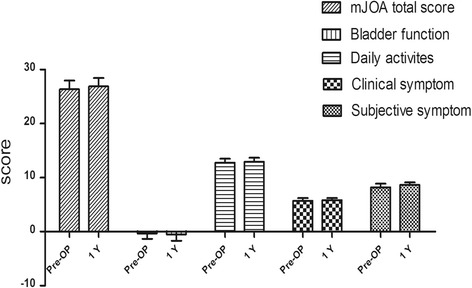
mJOA-score pre-operative (Pre-OP) and at the 1-year follow-up evaluation (1Y) in scoliosis patients with tethered spinal cords. * *P* > 0.05 vs. pre-operative

Neurologic function was assessed via the mJOA score. No significant difference existed between the pre-operative and follow-up evaluation total scores (*p* = 0.39). The pre-operative total score was 27 ± 2, and post-operatively the total score increased to 27 ± 2 at the 1-year follow-up evaluation. Subjective symptoms, clinical symptoms, daily activities and bladder function did not differ pre-operatively and at the follow-up evaluation (*p* = 0.07, *p* = 0.33, *p* = 0.44, *p* = 0.69; Table 4 and Fig. 6).

**Table 4:** mJOA score of preoperative and 1 year follow up

Parameters	Preoperative	1 year follow up	T value	*P* value
Subjective symptom	8 ± 1	9 ± 0.5	−1.95	0.07
Clinical symptom	6 ± 0.1	6 ± 0.4	−1.00	0.33
Daily activities	13 ± 1	13 ± 1	−0.82	0.44
Bladder function	−0.4 ± 1	−0.5 ± 1	0.44	0.69
mJOA total score	26 ± 2	27 ± 2	−0.88	0.39

(Note: Total mJOA-score was 29 including subjective symptom from 0 to 9 score, clinical symptom from 0 to 6 score, daily activities from 0 to 14 score and bladder function from −6 to 0 score.)

## Discussion

Spine deformities are always associated with spinal cord malformations [1, 14]. Common spinal cord malformations include diastematomyelia (types I and II), tethered spinal cords, syringomyelia, a low conus medullaris, spinal meningoceles, and lipomas [1, 15–17]. Multiple malformations can exist in an individual patient. In this study, nine of fourteen (64%) congenital scoliosis patients are associated with additional anomalies, including three patients (21%) with spina bifida occulta, three patients (21%) with vertebral deformity, two patients (14%) with syringomyelia, and one patients (7%) with ribs deformity. Of these, two patients (14%) had multiple abnormalities, one is associated with syringomyelia and polycystic ovary syndrome, and the other is associated with spina bifida occulta, syringomyelia and ribs deformity. It has been shown that 47% of pediatric tethered spinal cord patients had congenital or juvenile scoliosis [17, 18].

Most patients with tethered cord can be identified in patients with abnormality of the skin of the lumbar and sacral region and/or neurological dysfunction. However, some patients exhibit only scoliosis, which can also be considered as the first symptom of a tethered cord syndrome, without neurologic dysfunction at an early age [14]. The neurologic deficits which arise in these patients in adolescence or later life are caused by other factors, such as spinal stenosis, trauma over time, and increased physical activity [19]. These patients can be considered to have asymptomatic tethered cords.. While these patients had severe spinal deformity and/or scoliosis progression, a surgical correction is imperative for them.

TCS is usually diagnosed in childhood and a aggressive treatment to children with TCS is reasonable because they would have severe pain and advanced neurological deficits in the future [4]. For scoliosis patients with symptomatic tethered spinal cords (TCS), surgical detethering has consistently been reported to be effective in treating symptomatic tethered cord syndrome [10, 11]. There are three main opinions for treating scoliosis with tethered spinal cords. First, untethering is performed during the first-stage and second-stage posterior scoliosis correction is followed 3–6 months later. For slow progressive scoliosis and non-severe scoliosis, some authors stated that an early untethering procedure can prevent further progression of scoliosis [18] and can clearly relieve the symptoms of back pain and neurologic dysfunction [20]. They stated a surgical correction procedure can lead to excessive traction on the conus medullaris and spinal cord because of a degenerated thickened filum fixes the conus medullaris to he spinal canal, which increases the risk of neurologic injury and greatly increases the operative difficulty [17, 21, 22]. Complications, such as cerebrospinal fluid leakage and neurologic deterioration, are also common [23]. Second, some studies have suggested that surgical detethering and scoliosis correction can be performed at the same time and the risk of complications is not increased [12, 13]. This approach reduces the economic and mental burden of patients without the trauma of a second surgical procedure. Additionally, the surgical complications were decreased compared with a two-stage operation [24]; however, this procedure requires neurosurgeon and spine surgeon collaboration, which means longer operative time, increased intra-operative blood loss, a longer recovery time. Moreover, this approach cannot avoid untethering complications and a recurrent tethered spinal cord [25]. Third, a vertebral column resection (VCR) or spine shortening osteotomy have been shown to be safe and effective methods for treating scoliosis with spinal cord malformations, and are widely used in spine surgery [26–28]. A VCR or spine shortening osteotomy is especially applicable for a tethered spinal cord with severe kyphosis or kyphoscoliosis caused by a hemi-vertebra [29].

Whether a aggressive treatment and prophylactic untethering are reasonable and necessary are still controversial for school-aged children, adolescent patients with spinal cord who are neurologically intact [4, 14]. Some authors considered that releasing the tethered spinal cord may stabilize or even reverse the progression of scoliosis. A corrective procedure may cause irreversible neurological deficits unless untethering of the spinal cord has been performed previously [30]. A prophylactic untethering for an asymptomatic tethered spinal cord can reduce the traction of the spinal cord and avoid new neurologic dysfunction in a correction process. However, some authors had raised objections. They considered that a prophylactic untethering may cause post-operative complications and an uncertain long-term curative effect. Gharedaghi et al. [31] reported that those patients with early surgical untethering had the best results at the early follow-up evaluation, but the prognosis was nearly the same in both with or without untethering groups at a longer follow-up (> 3 years). Samdani et al. [32] also reported that a one-stage scoliosis correction without untethering is a safe and valuable approach for scoliosis patients with asymptomatic tethered spinal cords. Some authors also stated that school-aged children, adolescent and young adult patients have relatively advanced neurologic deficits, and the functional zone they have retained is very narrow, which means that it is necessary to maintain the balance between skeletal maturation and conus traction upon the TC. A aggressive treatment and prophylactic untethering could also result in a worsening of their neurologic deficits [14]. In this study, all the patients are school-aged children and adolescents with mean Risser’s score 4, (range: 9–17 years), which is a very critical age with spine growth and scoliosis correction. At this stage, they have limited growth potential and the cord tethering is unlikely to cause much problems. In these patients, 9 of 17 cases exhibited a scoliosis progression. Although it cannot be confirmed whether a tethered spinal cord induced a progressive scoliosis, a surgical correction was imperative. As such, one-stage posterior scoliosis correction without a prophylactic untethering were performed to improve deformity and stop curve progression. Based on our study, we further proved that in the school-aged children and adolescents with scoliosis combined with tethered cord, one-stage posterior scoliosis correction can be used as a surgical strategy for scoliosis patients with asymptomatic tethered spinal cords. A prophylactic untethering may be an appropriate procedure only when they had neurological symptoms.

For scoliosis combined with tethered cords, the most important issue is to maintain the balance between correction rate and stability of neurological status. Usually, one-stage posterior correction for treating scoliosis without untethering may cause neurologic injury or deterioration of tethered spinal cord symptoms. In this study; suspension radiograph, supine bending radiograph and fulcrum bending radiograph were used to evaluate the flexibility and expected correction rate, which can be partly considered as neural pliability for intraoperative correction. As a result, spine suspension, supine bending and fulcrum bending radiograph examination showed no apparent symptoms of a tethered spinal cord, which can be considered as there was no apparent changes of neurological status. Depending on these examinations, a better operative correction rate was achieved eventually. Thus, we suspected that the efficiency of a one-stage correction without untethering may be related to the negative results of spine suspension, supine bending and fulcrum bending radiograph examinations. Based on this study, we infer that while the flexibility of major curve was more than 30% in addition with the correction rate of fulcrum radiograph was more than 30%, it means that patients with scoliosis combined with tethered cord have a better neural function reserve, one-stage posterior correction is safe and effective without a prophylactic untethering.

Furthermore, whether a spinal cord might be changed is also concerned during the correction of a spinal deformity. If there exist some changes of spinal cord after scoliosis correction, then surgeons have to pay more attention to this phenomenon because this a correction procedure can cause some changes of the neurological status, which can lead to possible new symptoms and spinal cord injury. A study from Jae-Young Hong et al. [33] found that the conus medullaris level changed postoperatively in patients with severe scoliosis, and the spinal cord level changed after a large amount of correction. They also stated that the patients with scoliosis or lordosis had longer spinal column length to cord ratios, which meant a relative stretching of the cord. If the scoliosis or lordosis is corrected, then the length of spinal column can be decreased, following the decrease of the upward tethering force. In this study, all the patients were mild to moderate scoliosis (the cobb angle of major curve is less than 90 degrees), which means a limitation of correction, the conus medullaris probably will not be changed after a correction procedure, which means no excessive traction during correction procedure and after surgery.

Admittedly, there were several limitations in our research. First, the number of cases was small and the duration of follow-up was short. Whether or not long-term symptoms will arise warrants further observation. Second, for patients with severe scoliosis with a coronal Cobb angle >90^o^, severe kyphosis and kyphoscoliosis, whether or not this approach is applicable also warrants further study. Third, the indication for one-stage correction without untethering has some limitations.

## Conclusion

Our study demonstrated that, a one-stage posterior scoliosis correction did not increase the risk of immediate neurologic dysfunction in scoliosis patients with tethered spinal cords. A one-stage posterior correction without a prophylactic untethering can possibly be a safe and valuable surgical approach if the patients had adequate neurologic reserve. However, study limitations need to considered and further study is warranted.

## Abbreviation

AVR: Apical vertebral rotation
AVT: Apical vertebral translation
CR: Correction rate
CT: Three-dimensional computed tomography
L: Lumbar
MEP: Motor evoked potentials
mJOA: Modified Japanese Orthopaedic Association
MRI: Magnetic resonance imaging
MT: Major thoracic
PT: Proximal thoracic
SEP: Somatosensory evoked potentials
SRS: Scoliosis Research Society
TC: Tethered spinal cord
TCS: Tethered cord syndrome
TL: Thoracolumbar
VCR: Vertebral column resection
